# Efficient removal of Remazol Red dye from aqueous solution using magnetic nickel ferrite nanoparticles synthesized via aqueous reflux

**DOI:** 10.1038/s41598-025-98478-y

**Published:** 2025-05-20

**Authors:** Ahmed Anwar Hassan, Mohamed Eid M. Ali, Samir A. Abdel-Latif, Ibrahim W. Hasani, Yosri A. Fahim

**Affiliations:** 1 Egyptian Mineral Resources Authority (EMRA) and ISK Gold Lab, Cairo, Egypt; 2 Water Pollution Research Department, National Research Centre, Dokki, Cairo, 12622 Egypt; 3 Department of Chemistry, Faculty of Science, Helwan University, Cairo, 11795 Egypt; 4 Department of Pharmaceutics, Faculty of Pharmacy, S.P.U., M.P.U and Idlib University, Idlib, Syria; 5 Health Sector, Faculty of Science, Galala University, Galala City, Suez, 43511 Egypt

**Keywords:** Surface adsorption, Nickel ferrite, Wastewater treatment, Remazol red dye, Environmental sciences, Chemistry

## Abstract

Rapid growth of the textile industry, along with the excessive use of water and dyes, has led to significant environmental concerns. This study introduces a straightforward, low-temperature aqueous reflux method for the fabrication of magnetic nickel ferrite (NiFe_2_O_4_) nanoparticles. The synthesized nanoparticles, characterized by X-ray diffraction (XRD), Fourier-transform infrared (FTIR) spectroscopy, scanning electron microscopy (SEM), and vibrating sample magnetometry (VSM), exhibited a cubic spinel structure, an average particle size of 23 ± 2.3 nm (range: 18–29.8 nm), and a magnetization of 56.96 ± 0.9 emu/g, enhancing their surface area and magnetic separability. These NiFe_2_O_4_ nanoparticles achieved a 96.5 ± 0.4% removal efficiency of Remazol Red dye from aqueous solutions after 90 min, with an adsorption capacity (q_max_) of 169.5 ± 0.8 mg/g, as tested across pH 2–12, contact times of 10–120 min, and initial dye concentrations of 20–200 mg/L. Optimal removal occurred at pH 2, with a dye concentration of 20 mg/L and a 1 g/L dose, yielding 99 ± 0.5% efficiency, while adsorption decreased at high pH due to surface charge effects (PZC = 6.7). The results indicated that dye adsorption increased with decreasing pH and higher nickel ferrite dosage. Kinetic studies over 10–120 min followed pseudo-first-order (R^2^ = 0.96), Boyd, and Weber–Morris models, while isotherms across 20–200 mg/L conformed to the Freundlich model (R^2^ = 0.98), reflecting multilayer adsorption. These properties high crystallinity, nanoscale size, and strong magnetic responsiveness enhance the material’s surface area, adsorption capacity, and ease of separation, contributing to its efficiency as an adsorbent. Reusability tests confirmed the stability of the nanoparticles and their consistent performance across multiple cycles. These results establish NiFe_2_O_4_ as an economical, magnetically separable, and ecologically sustainable adsorbent for wastewater treatment purposes.

## Introduction

The contamination of wastewater with xenobiotic and hazardous pollutants is a significant concern for governments.^1,2^ Pollutants like heavy metals and dyes have a severe impact on the environment and pose substantial risks to human health, highlighting the pressing need for effective remediation strategies.^3^ Addressing this challenge is essential for protecting ecosystems and ensuring public health. There is an urgent demand for creative and eco-friendly treatment options. Prompt intervention is crucial to reduce risks and safeguard water resources.^4^ The textile industry’s wastewater is the principal source of carcinogenic and mutagenic chemicals that harm aquatic life.^5^ Azo dyes are a substantial category of synthetically manufactured dyes that may induce genetic alterations and carcinogenesis.^6^ The presence of these compounds in wastewater causes the pollution of water resources.^7^ Extensive efforts have been undertaken to eliminate these hazardous dyes from industrial effluents. However, the removal of azo dyes from industrial wastewater remains highly challenging ^8^. Activated sludge, coagulation, and flocculation methods are frequently employed in wastewater treatment.^9^ Unfortunately, aerobic biological treatment fails to degrade the azo double bonds in reactive dyes, leading to persistent effluent coloration.^10^ While the coagulation process can partially decolorize materials, the sludge generated from both processes is challenging to manage and remove.^11^ Adsorption is recognized as an efficient and affordable technology. It offers a cost-effective solution for removing contaminants from gases and liquids.^12,13^ Currently, nanotechnology applications are extensively used for pollutant removal, with magnetic adsorbents being employed for wastewater treatment ^14^. Nanoparticles are small particles with at least one dimension inside the nanoscale range, less than 100 nm.^15,16^ Ferrite nanoparticles have garnered significant interest as adsorbents due to their remarkable adsorption capability, extensive surface area, affordability, ease of production, and environmental sustainability.^17^ Spinel ferrites serve as effective adsorbents in water purification processes, facilitating the removal of hazardous pollutants from aquatic environments.^18^ The growing interest in magnetic ferrite nanoparticles arises from their diverse applications in permanent magnets, microwave devices, and high-density information storage technologies.^19^ Nickel ferrite is widely recognized as a technologically important soft ferrite material due to its outstanding ferromagnetic properties, low conductivity, minimal eddy current losses, and excellent electrochemical stability.^20^

Due to their magnetic separability and adsorption capabilities, nickel ferrite-based materials have garnered significant attention for their efficacy in dye removal from wastewater. Research has shown their effectiveness in eliminating dyes like Congo Red and Methylene Blue, utilising their extensive surface area and magnetic characteristics for excellent pollutant sequestration and retrieval.^18,21^ Fathy et al. reported the application of nickel ferrite nanoparticles modified with poly(aniline-*co*–o-toluidine) to eliminate 2,4-dichlorophenol, attaining substantial adsorption owing to improved surface functionality.^21^ Similarly, Taj et al*.* used bioconjugated nickel ferrite nanoparticles to eliminate Congo Red, underscoring their efficacy in addressing dye-polluted water.^22^ Challenges related to nickel ferrite-based materials encompass restricted adsorption capacity resulting from nanoscale agglomeration, diminished stability throughout many cycles, and the necessity for high-temperature synthesis techniques that undermine cost-effectiveness and scalability.^17,23^ The conventional solid-state reaction method for producing nickel ferrite requires a high calcination temperature, resulting in inhomogeneity, poor stoichiometry, and larger crystallite sizes.^23^ Various chemical methods have been used in the past to produce nanocrystalline NiFe_2_O_4_, including ultrasonication-assisted synthesis, co-precipitation, sol–gel, microwave, reverse micelle, and hydrothermal processes.^17,24^ The characterization and synthesis of magnetic nanoparticles responsive to magnetic fields garner considerable interest due to their remarkable potential in various applications.^25^

In this study, we selected the aqueous reflux method for synthesizing NiFe_2_O_4_ nanoparticles due to its simplicity, cost-effectiveness, and ability to produce well-controlled morphology and phase-pure nanoparticles under mild conditions. Compared to the co-precipitation method, which often requires post-synthesis calcination steps that may affect particle size and surface properties, the aqueous reflux technique allows for better control over crystallinity without high-temperature post-processing. Similarly, hydrothermal synthesis, though effective, requires high pressures, making it less accessible and scalable than the aqueous reflux approach. Our method ensures the formation of highly crystalline NiFe_2_O_4_ nanoparticles suitable for adsorption applications while maintaining ease of synthesis and scalability. This approach allows for the production of the desired product without the need for calcination, while maintaining precise control over reaction parameters. The synthesized magnetic nickel ferrite nanoparticles were then utilized for the efficient removal of Remazol Red dye from aqueous solutions through an adsorption process.

## Materials and methods

### Materials

The following reagent-grade materials were employed in the study, sourced from commercial suppliers: Hydrated Ferric Chloride (FeCl_2_·6H_2_O, 96%) and Nickel Chloride (NiCl_2_·6H_2_O, 98%) were obtained from ADVENT Chem Bio, while Ammonium hydroxide (NH_4_OH, 25%), Hydrochloric acid (HCl, 35%), and Remazol Red dye (98%) were procured from Alpha Chemika. All chemicals were utilized without further purification. All experiments were conducted with ultrapure Milli-Q water.

### Nickel ferrite nanoparticle (NiFe_2_O_4_) synthesis

Magnetic Nickel Ferrite was synthesized by an aqueous reflux method building on techniques such as those reported in ^26^, adapted in this study for NiFe_2_O_4_ with a low-temperature, calcination-free process. A precursor solution was prepared by dissolving nickel chloride (NiCl_2_·6H_2_O) and hydrated ferric chloride (FeCl_2_·6H_2_O) in deionized water at a 1:2 molar ratio. Subsequently, a 25% ammonia solution was introduced dropwise while continuously monitoring the pH until it reached 9.5. The resulting solution was vigorously stirred using a magnetic stirrer in a condensation and reflux device. The mixture was heated to its boiling point and refluxed for two hours for a homogeneous reaction and suitable synthesis conditions.

**Fig. 1:**
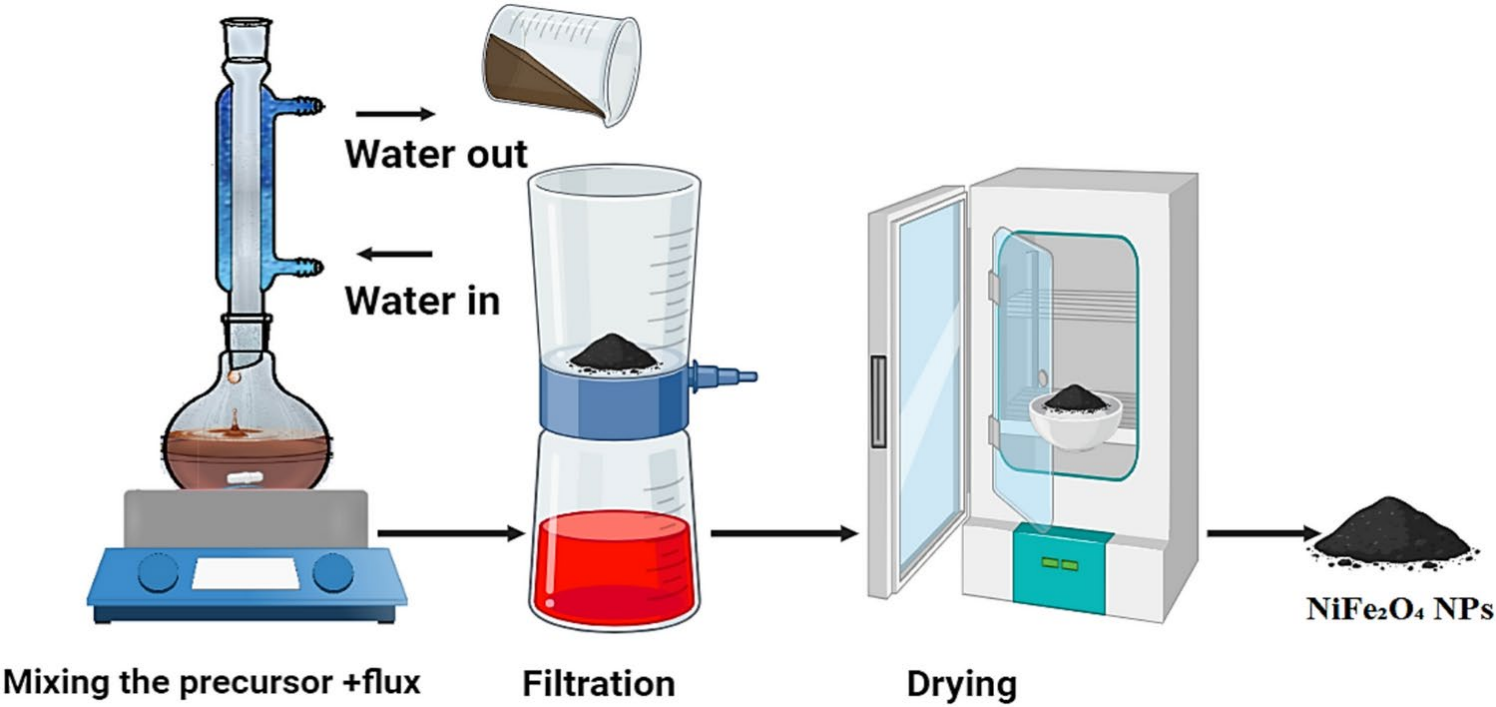
Aqueous reflux synthesis of magnetic nickel ferrite NPs.

To reduce the agglomeration of the synthesized NiFe_2_O_4_ nanoparticles, the addition of ammonia was meticulously regulated to facilitate slow nucleation and development, hence averting fast particle clustering. The vigorous agitation during refluxing enhanced the uniform dispersion of nanoparticles in the solution, diminishing the potential for agglomeration. Preliminary experiments were conducted to evaluate the influence of key reaction parameters, including reaction temperature, reflux duration, and precursor concentration. This process facilitated the formation of nickel ferrite (NiFe_2_O_4_) nanoparticles, which were obtained as a dark-colored solid exhibiting high magnetic responsiveness. The nanoparticles were meticulously rinsed three times with distilled water to remove residual contaminants and unreacted precursors, resulting in a pure product appropriate for subsequent characterization and applications, as shown in Fig. 1.

### Characterization of synthesized magnetic nickel ferrite NPs

#### X‑ray diffraction (XRD)

The phase structure was analysed at room temperature using a Phillips X’Pert PRO X-ray diffraction (XRD) apparatus, which employed Cu Kα radiation at 50 kV and 40 mA. A secondary beam graphite monochromator was utilized to enhance measurement precision. Diffraction patterns were recorded over a 2θ range of 20° to 60°, with a step size of 0.02° and a measurement time of 2 s per step.

#### Fourier transform infrared spectroscopy (FTIR)

Fourier transform infrared (FTIR) spectroscopy was utilized to analyze the functionalized surface of nickel ferrite nanoparticles. The analysis was performed using an Agilent Technologies Cary 630 FTIR spectrometer. The spectra were acquired over a range of 4000–400 cm^–1^ with a resolution of 4 cm^–1^, averaging 32 scans per measurement.

#### Scanning electron microscopy (SEM)

Scanning Electron Microscopy (SEM) is a rapid and precise technique for analyzing materials’ surface morphology and microscopic properties. This work utilized scanning electron microscopy (SEM) imaging and energy-dispersive X-ray spectroscopy (EDX) analysis conducted with a TESCAN VEGA COMPACT SEM, which included a tungsten filament as the electron source. The EDX detector was included in a JEOL JSM-6510 LV SEM microscope located in Kohoutovice, Czech Republic.

#### Vibrating sample magnetometer (VSM)

The magnetic characteristics were assessed at ambient temperature utilizing a Lakeshore 7410 vibrating sample magnetometer (VSM) with an applied magnetic field of 20 kOe. The magnetization characteristics of the synthesized magnetic nanoparticles exhibited a characteristic hysteresis loop, indicating distinct remanent magnetization and coercivity values.

#### Adsorption studies

**Fig. 2:**
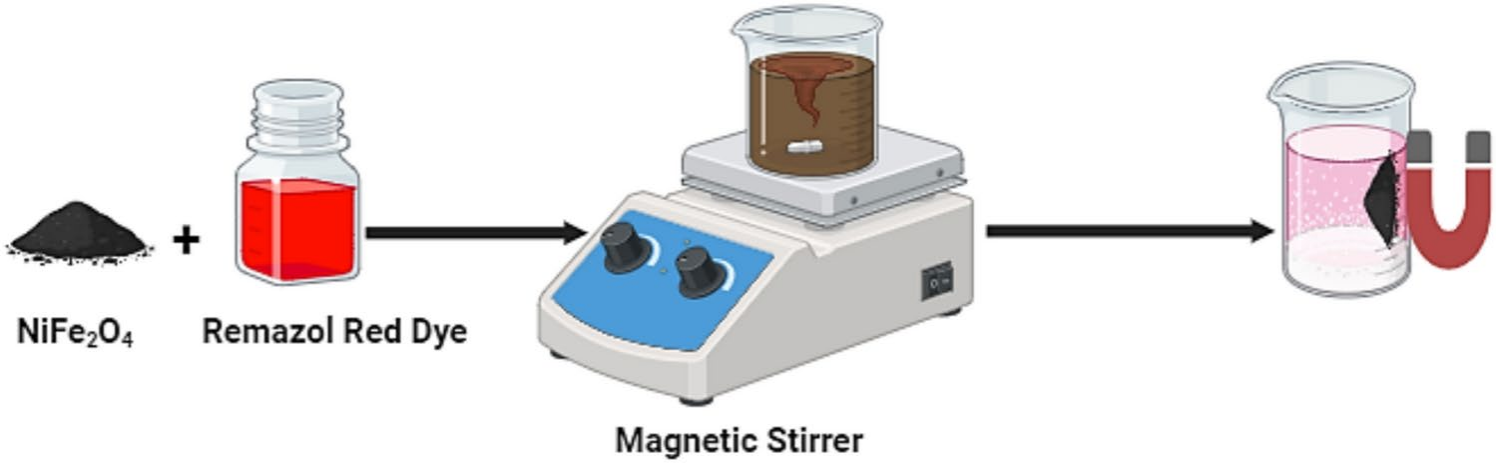
Adsorption of Remazol Red dye on Synthesized Magnetic NiFe_2_O_4_ NPs.

Adsorption experiments were conducted by introducing varying amounts of nickel ferrite into conical flasks (100 mL) containing Remazol Red dye solutions of different concentrations and pH levels. The pH adjustments were made using either hydrochloric acid (HCl) or sodium hydroxide (NaOH). The mixtures were stirred at a constant temperature of 25 °C for varying time intervals, following which the solid and liquid phases were separated by magnetic separation, as illustrated in Fig. 2. The residual concentration of Remazol Red dye in the filtrate was then determined using a UV–Vis spectrometer at a wavelength of 540 nm ^27^. The removal efficiency % was calculated based on the following equation:

1 $$\mathbf{Removal~efficiency\%}=\frac{{{\mathbf{C}}_{0}-\mathbf{C}}_{\mathbf{e}}}{{\mathbf{C}}_{0}}\times 100$$

where C_o_ and C_e_ represent to the initial and final concentrations of Remazol Red dye in the solution, respectively.

The adsorption kinetics were evaluated by experiments employing an initial dye concentration of 20 mg/L, a nickel ferrite dosage of 1 g/L, a temperature of 25 °C, and a pH of 6. Samples were taken at regular intervals, and the concentration of Remazol Red dye was measured. The following kinetic models were applied:

**Pseudo-first-order model:**

Which is given as the following equation:^28^

2 $$\mathbf{ln}\left({{\varvec{q}}}_{{\varvec{e}}}-{{\varvec{q}}}_{{\varvec{t}}}\right)=\mathbf{ ln}{{\varvec{q}}}_{{\varvec{e}}}-{{\varvec{K}}}_{1}{\varvec{t}}$$

In this context, q_e_ and q_t_ signify the quantity of Remazol Red adsorbed on the sorbent (mg/g) at equilibrium and at a certain time t, respectively, whereas K_1_ denotes the rate constant for first-order adsorption (min^−1^).

**Pseudo-second-order model**

Which is represented as the following equation:^29^

3 $$\frac{{\varvec{t}}}{{{\varvec{q}}}_{{\varvec{t}}}}=\frac{{\varvec{t}}}{{{\varvec{q}}}_{{\varvec{e}}}}+\frac{1}{{{\varvec{k}}}_{2}{{{\varvec{q}}}_{{\varvec{e}}}}^{2}}$$

where q_t_ and qe represent the quantity of material adsorbed on NiFe_2_O_4_ at time t and equilibrium (mg/g), respectively, the second-order adsorption rate constant, K_2_, is expressed in g/mg min. The linear plots of t/q_t_ against time were examined to ascertain the rate parameters.

**Weber–Morris kinetic model**

Which is mathematically expressed as follows:^30^

4 $${{\varvec{q}}}_{{\varvec{t}}}={{\varvec{k}}}_{{\varvec{d}}}{{\varvec{t}}}^{1/2}+{\varvec{I}}$$

The parameter K_d_ denotes the rate constant for intra-particle diffusion, whereas the I values provide valuable information regarding the boundary layer thickness ^31^.

**Boyd model**

Which is expressed in the following equation.^32^

5 $${{\varvec{B}}}_{{\varvec{t}}}=-\mathbf{ln}\left({\bf 1}-\frac{{{\varvec{q}}}_{{\varvec{t}}}}{{{\varvec{q}}}_{{\varvec{e}}}}\right)-\mathbf{0.4977}$$

where B_t_ is a function of the fractional attainment of equilibrium. To study the adsorption isotherms, the experimental data obtained were fitted to the following isotherm models:

**Langmuir isotherm model**

The Langmuir equation is expressed in the following equation:^33,34^

6 $$\frac{{{\varvec{c}}}_{{\varvec{e}}}}{{{\varvec{q}}}_{{\varvec{e}}}}=\frac{1}{{{\varvec{k}}}_{{\varvec{L}}}{{\varvec{q}}}_{{\varvec{m}}{\varvec{a}}{\varvec{x}}}}+\frac{{{\varvec{c}}}_{{\varvec{e}}}}{{{\varvec{q}}}_{{\varvec{m}}{\varvec{a}}{\varvec{x}}}}$$

In this context, q_max_ denotes the maximal monolayer adsorption (mg/g), while K_L_ signifies the Langmuir constant related to the affinity of binding sites (L/mg).

**Freundlich isotherm model**

The Freundlich equation, which characterizes multilayer adsorption, is expressed by the subsequent formula:^35^

7 $${\varvec{ln}}{{\varvec{q}}}_{{\varvec{e}}}={\varvec{ln}}{{\varvec{k}}}_{{\varvec{F}}}+\frac{1}{{\varvec{n}}}{\varvec{ln}}{{\varvec{c}}}_{{\varvec{e}}}$$

where K_F_ is the Freundlich adsorption capacity (mg/g) and 1/n, correspond to the adsorption intensity.

**Temkin isotherm model**

The Temkin isotherm assumes that sorption heat diminishes linearly with increasing sorption coverage on the adsorbent.^36^ Temkin isotherm equation is expressed as:^37^

8 $${{\varvec{q}}}_{{\varvec{e}}}={{\varvec{B}}}_{{\varvec{T}}}\boldsymbol{ln}{{\varvec{k}}}_{{\varvec{T}}}+{{\varvec{B}}}_{{\varvec{T}}}\boldsymbol{ln}{{\varvec{c}}}_{{\varvec{e}}}$$

where B_T_ is related to the heat of adsorption and K_T_ is the Temkin constant.

**Dubinin–Radushkevich isotherm model**

The Dubinin–Radushkevich (D–R) isotherm was utilized to ascertain the nature of the chemical or physical adsorption mechanism. The Dubinin–Radushkevich isotherm is articulated as follows:^38^

9 $${\varvec{l}}{\varvec{n}}{{\varvec{q}}}_{{\varvec{e}}}={\varvec{l}}{\varvec{n}}{{\varvec{q}}}_{{\varvec{D}}{\varvec{R}}}-{\varvec{\beta}}{{\varvec{\varepsilon}}}^{2}$$

where q_DR_ refers to the Dubinin–Radushkevich monolayer capacity (mol/g), β is the adsorption energy constant (mol^2^/J^2^), and ε represents the Polanyi potential, which is associated with the equilibrium concentration as follows:

10 $${\varvec{\varepsilon}}={\varvec{R}}{\varvec{T}}{\varvec{l}}{\varvec{n}}\left(1+\frac{1}{{{\varvec{C}}}_{{\varvec{e}}}}\right)$$

The D-R isotherm parameter β is utilized to ascertain the adsorption energy (E) in kJ/mol, as detailed below:

11 $${\varvec{E}}=\frac{1}{\sqrt{2{\varvec{\beta}}}}$$

## Results and discussion

### UV–Vis spectroscopy of Remazol Red dye

**Fig. 3:**
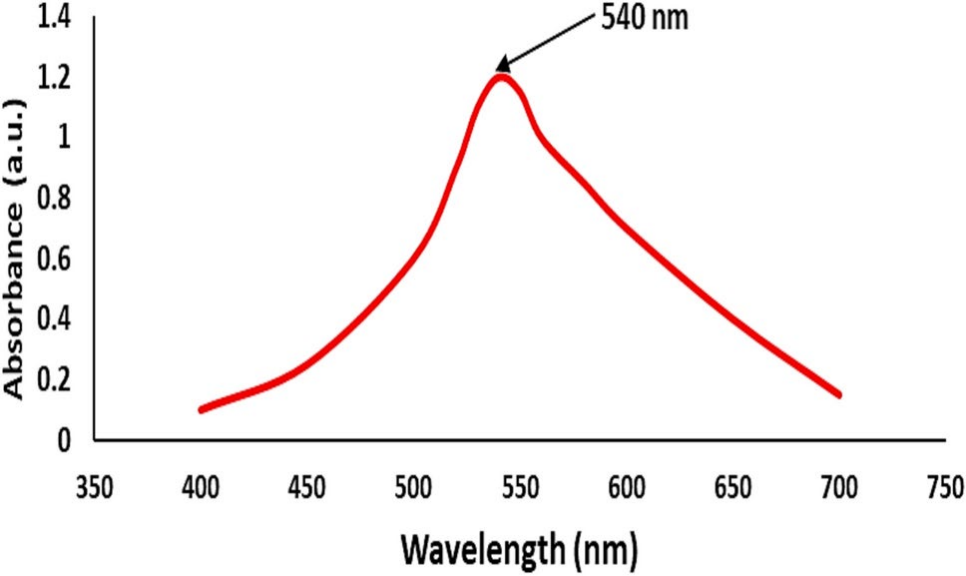
UV–Vis absorption spectrum of Remazol Red dye in aqueous solution.

The UV–Vis absorption spectrum of Remazol Red dye was analysed to verify the wavelength employed for concentration measurements in adsorption investigations. Figure 3 illustrates that the spectrum, obtained at 25 °C in aqueous solution, has a significant peak at 540 nm, therefore designating this wavelength as the maximum absorbance (λmax). This finding corresponds with other previous studies.^39,40^

### Characterization of synthesized NiFe_2_O_4_

#### X‑ray diffraction (XRD)

**Fig. 4:**
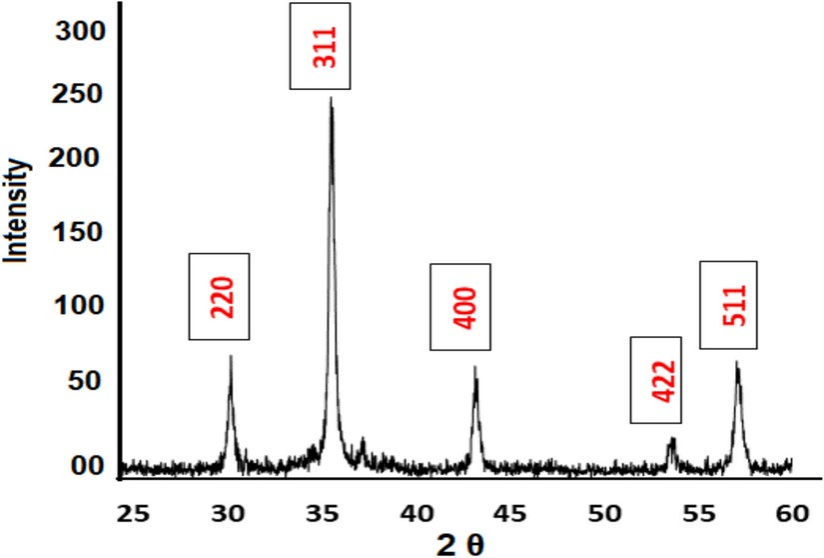
XRD of synthesized magnetic NiFe_2_O_4_ NPs.

Figure 4 illustrates the XRD patterns of NiFe_2_O_4_ nanoparticles produced by the aqueous reflux method. The XRD analysis confirmed the formation of a cubic spinel structure, with distinct diffraction planes clearly identifiable. The observed diffraction peaks were in close agreement with the standard pattern provided in the JCPDS database (Card No. 10-325).^41^ This confirms the effective synthesis of phase-pure nickel ferrite. The prominent diffraction peaks were observed at 2θ angles of 30.15°, 35.6°, 43.3°, 53.3°, and 57.23°, corresponding to the (220), (311), (400), (422), and (511) crystal planes, respectively. These planes align with the typical spinel structure of NiFe_2_O_4_, further validating the material’s crystallographic integrity.^21^ The well-defined and sharp peaks indicate high crystallinity in the synthesized nanoparticles, a characteristic often associated with uniform particle size and effective synthesis methods. Additionally, the XRD pattern of the synthesized nickel ferrite closely resembles patterns reported in previous studies, including those by Huo and Wei.^42^ This consistency highlights the reproducibility and reliability of the aqueous reflux method in formation of nickel ferrite with a well-defined cubic spinel structure.

#### Fourier transform infrared spectroscopy (FTIR)

**Fig. 5:**
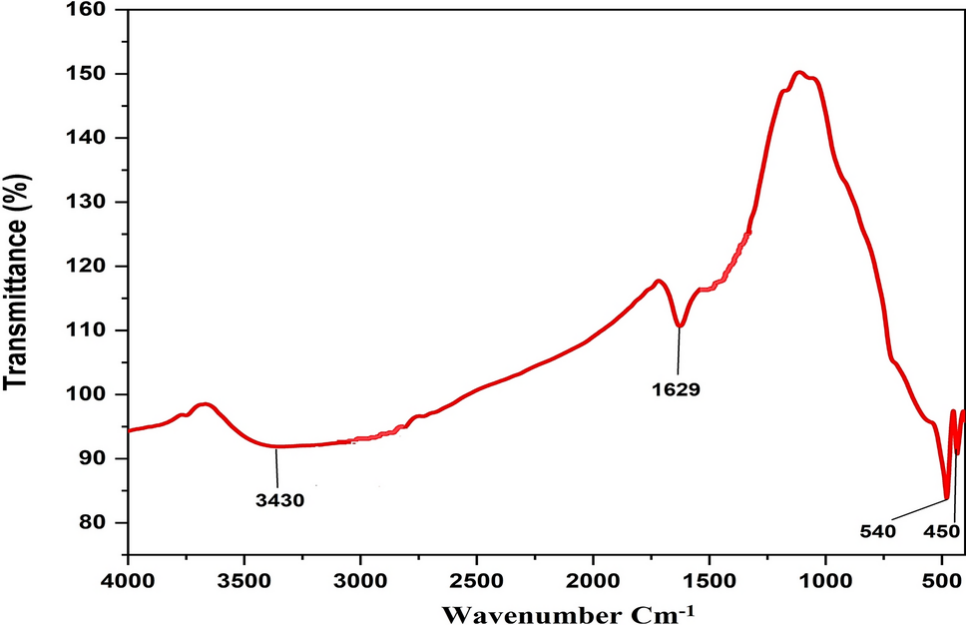
FTIR spectrum of synthesized magnetic NiFe_2_O_4_ NPs.

Fourier Transform Infrared (FTIR) spectra were acquired in the wavelength range of 4000–400 cm^–1^ to confirm the composition of the synthesized ferrite. Figure 5 illustrates the FTIR spectrum of NiFe_2_O_4_. The two primary bands seen between 585 and 453 cm^–1^ are ascribed to the inherent stretching vibrations of Fe–O and Ni–O bonds inside the spinel structure.^43^ The absorption peak at 540 cm^–1^ corresponds to the Fe–O vibrational mode, whereas the peak at 450 cm^–1^ pertains to the Ni–O vibration ^44^. No notable peaks were seen below 400 cm^–1^ in the infrared (IR) spectrum. The peak at 1629 cm^–1^ is linked to O–H bending, whereas the wide peak at 3430 cm^–1^ pertains to the O–H stretching vibration of unbound or adsorbed water molecules.^45^

#### Scanning electron microscopy (SEM)

**Fig. 6:**
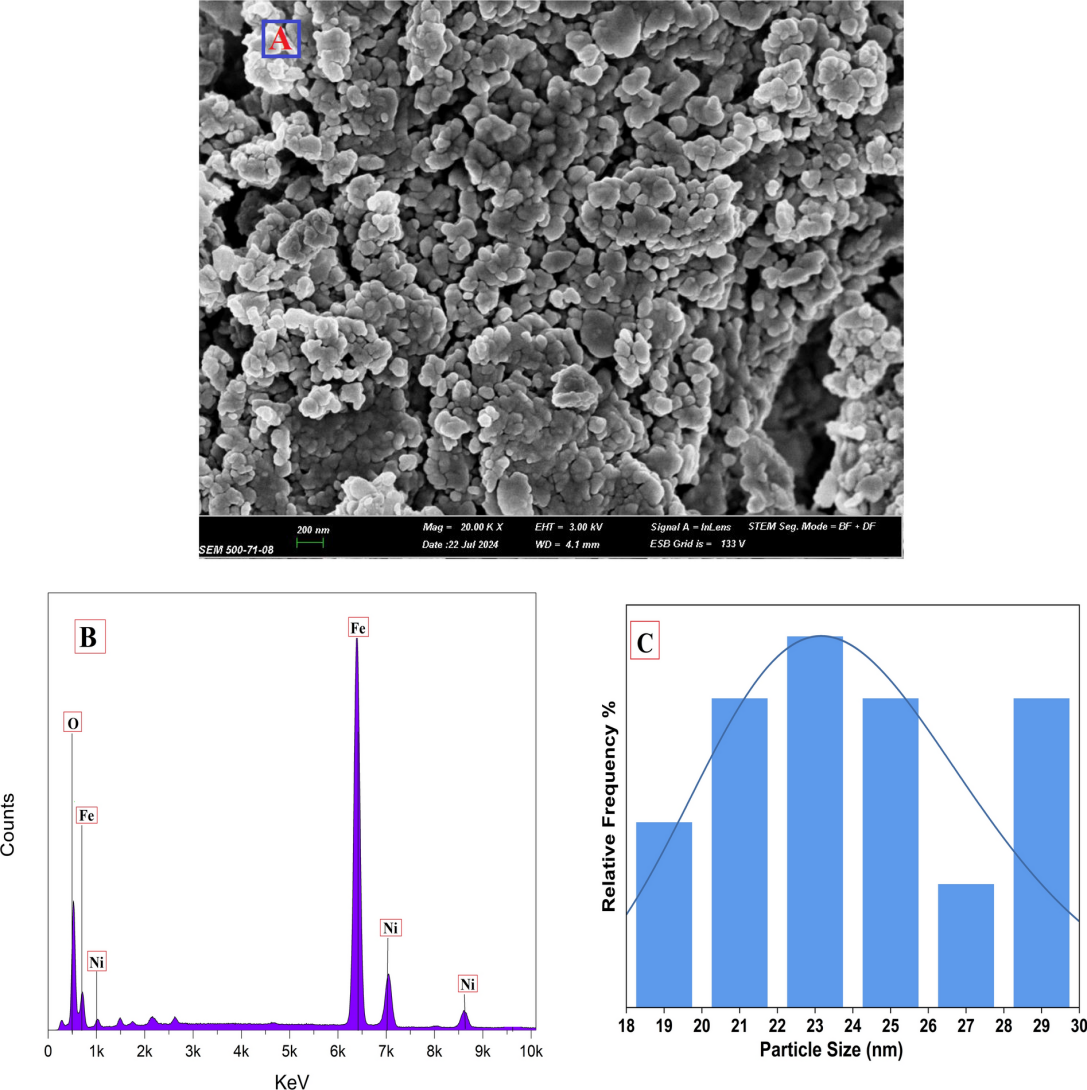
(**A**) SEM, (**B**) EDX and (**C**) particle size distribution images of synthesized magnetic NiFe_2_O_4_ NPs.

Figure 6 displays SEM and EDX pictures alongside the particle size distribution curve of NiFe_2_O_4_. The photos demonstrate a homogeneous distribution and unique shape of the synthesized magnetic NiFe_2_O_4_ nanoparticles, with particle diameters between 18 and 29.8 nm. The EDX analysis identified characteristic peaks corresponding to Fe, Ni, and O atoms, confirming the synthesis of NiFe_2_O_4_ nanoparticles. The average particle size and size distribution, as determined from the SEM analysis, is around 23 ± 2.3 nm. The studied particle size distribution demonstrates minor fluctuations attributable to several synthesis factors, such as precursor concentration, reaction temperature, reflux time, and pH level. These variables affect nucleation and growth kinetics, resulting in variations in particle size.

#### Magnetic characterizations

**Fig. 7:**
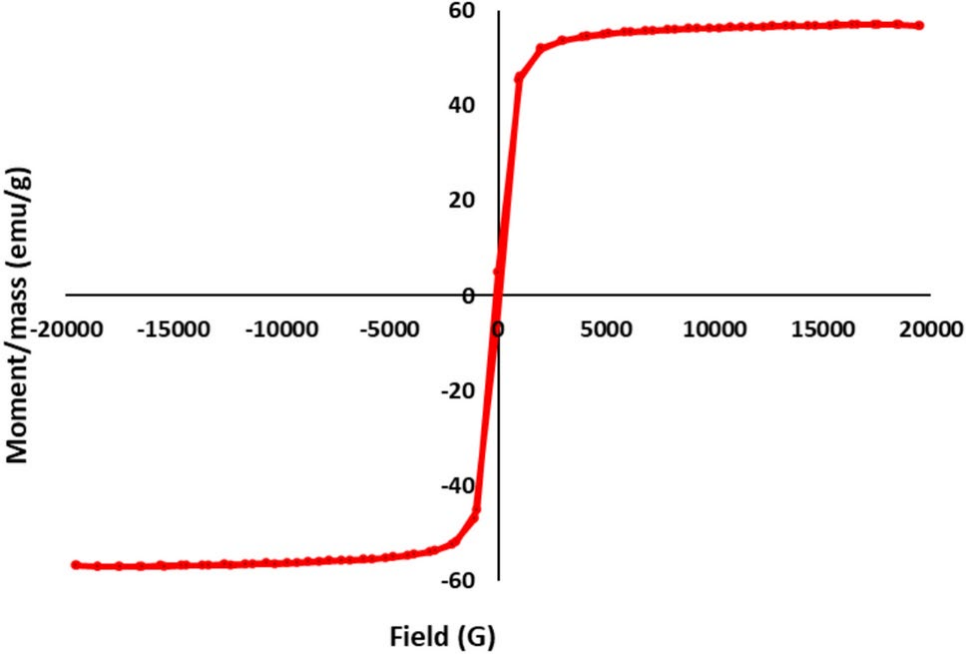
Vibrating sample magnetization of synthesized magnetic NiFe_2_O_4_ NPs.

Figure 7 illustrates the hysteresis loops of the synthesized NiFe_2_O_4_, highlighting the material’s magnetic properties. Magnetic nickel ferrite displayed a magnetization value of 56.96 emu/g, which is notably higher than the 55 emu/g reported for multidomain bulk nickel ferrite^46^. The increased magnetization suggests that the prepared nickel ferrite nanoparticles exhibit superparamagnetic behavior. Superparamagnetic materials are characterized by their ability to show strong magnetization when exposed to an external magnetic field, while retaining minimal remanence and coercivity once the field is removed^47^. In addition to its high magnetization, the synthesized nickel ferrite exhibited a low coercivity value of 92 G. Coercivity indicates the resistance of a magnetic material to changes in magnetization and is an important factor in assessing the material’s magnetic reversibility. The high magnetization and low coercivity observed in the prepared nickel ferrite can be attributed to its nanoscale size, uniform morphology, and high crystallinity^48^. These structural features reduce defects and improve the alignment of magnetic domains, resulting in enhanced magnetic properties. The excellent magnetic characteristics of the prepared nickel ferrite make it a promising material for a variety of applications, especially in areas such as magnetic separation, biomedical applications, and environmental remediation, where strong magnetization and easy magnetic reversibility are highly desirable^49^.

#### Point of zero charge

**Fig. 8:**
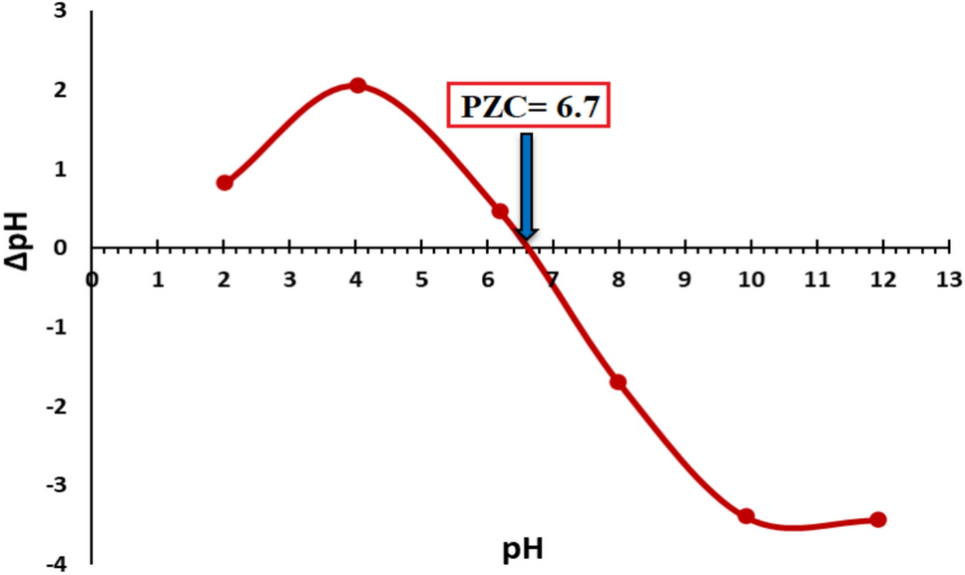
PZC of nickel ferrite NPs.

The point of zero charge (PZC) of NiFe_2_O_4_ was ascertained using the salt addition method by immersing the material in 0.01 mol/L NaCl solutions at room temperature (25ºC). In separate beakers, 50 mg of NiFe_2_O_4_ was combined with 50 mL of a 0.01 mol/L NaCl solution. The pH of the solution was modified to 2, 4, 6, 8, 10, and 12, utilizing HCl or NaOH as required. The pH values were recorded before sealing each flask with parafilm and subjecting them to continuous agitation for 24 h. The solutions’ final pH values were recorded, and the difference between the initial and final pH, also known as ΔpH, was graphed against the initial pH values^50^. The PZC values were calculated from ΔpH versus pH plots, at the pH where ΔpH = 0^51^. The curve intersects at the PZC, designated as 6.7, as seen in Fig. 8. The experimental results demonstrate that ferrite particles have a positive charge in acidic solutions. Moreover, at pH levels beyond the point of zero charge (PZC), the surface charge density of the ferrites progressively increases, leading to augmented electrostatic repulsion. Our study’s PZC results are consistent with previously reported values, which vary depending on the synthesis method, precursor materials, and surface modifications^22,52^.

### Adsorption studies on synthesized magnetic NiFe_2_O_4_ NPs

#### Effect of pH

**Fig. 9:**
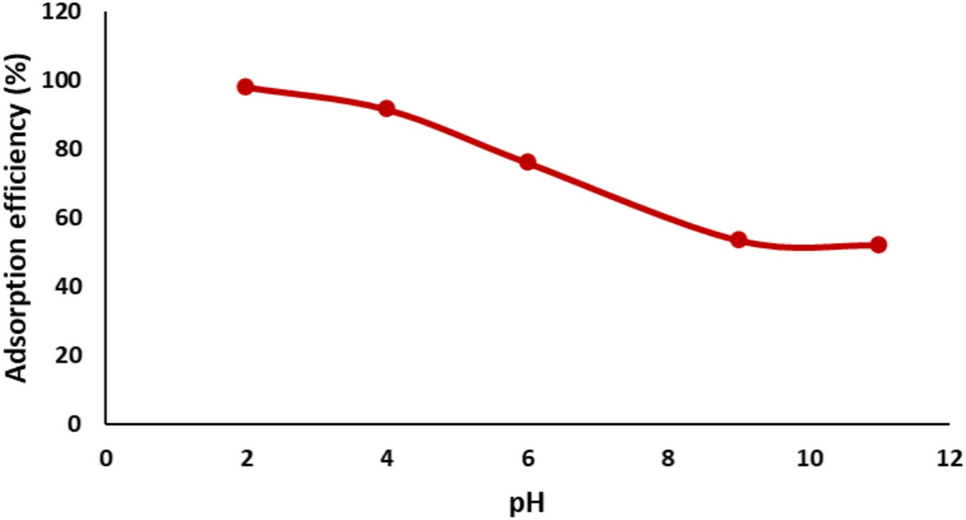
Impact of pH on the adsorption efficiency of Remazol Red dye.

pH measures the acidity or alkalinity of an aqueous solution. Understanding its influence is essential for studying the adsorption of dyes^53^. The experiment involved applying 50 mL of a solution containing Remazol Red dye at a concentration of 20 mg/L to assess the adsorption capabilities of nickel ferrite at various pH levels. The outcome is displayed in Fig. 9. The pH dramatically influences the degree of dye ionization and the surface properties of the adsorbents. The adsorption of Remazol Red dye on the surface of nickel ferrite reduced as the pH of the dye solution increased. The behavior of NiFe_2_O_4_ nanoparticles in adsorption can be explained by their point of zero charge (PZC), determined as 6.7 in this study. At a pH below 6.7, the nanoparticle surface acquires a positive charge due to protonation by H⁺ ions, hence augmenting the electrostatic interaction with the negatively charged sulfonic groups of Remazol Red, a reactive azo dye. This results in improved adsorption efficiency, with optimal dye removal observed at pH 2. Conversely, at pH above 6.7, deprotonation causes the NiFe_2_O_4_ surface to acquire a negative charge, leading to electrostatic repulsion with the anionic dye molecules and significantly reducing adsorption effectiveness. This pH-dependent adsorption trend, consistent with previous studies, aligns with the measured PZC of 6.7 for NiFe_2_O_4_^54,55^.

#### Effect of nickel ferrite dose

**Fig. 10:**
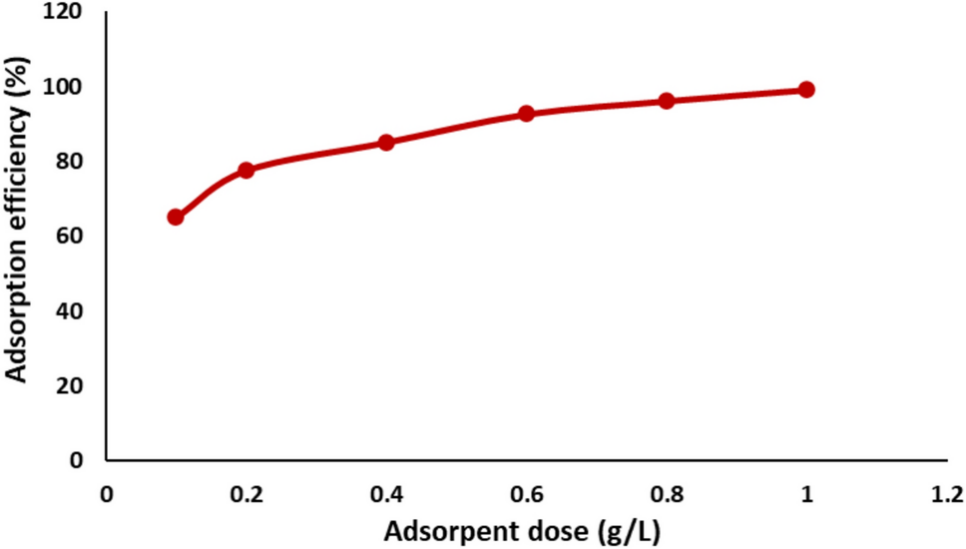
Effect of adsorbent dose on the adsorption efficiency of Remazol Red dye.

The adsorbent dosage is a crucial factor, as it significantly influences the efficiency of the treatment process^56^. To determine the optimal dosage of nickel ferrite for the effective adsorption of Remazol Red dye, a series of experiments were performed. Different quantities of nickel ferrite (ranging from 0.1 to 1 g) were introduced into flasks containing dye solutions with an initial concentration of 20 mg/L. The results, as shown in Fig. 10, revealed that as the amount of nickel ferrite increased from 0.1 g to 1 g, the adsorption efficiency improved significantly, rising from 65 ± 0.9 to 99 ± 1.1%. However, beyond a certain point, no substantial increase in the adsorption rate was observed. The enhancement in adsorption efficiency with elevated ferrite dosages is ascribed to the increased availability of active surface sites for dye molecules to adhere. Interestingly, the increase in removal efficiency from 0.8 to 1 g/L of nickel ferrite was marginal, with only a 3% improvement. This suggests that beyond 0.8 g/L, the adsorption process nears saturation, and additional adsorbent does not contribute significantly to further dye removal.

#### Effect of contact time

**Fig. 11:**
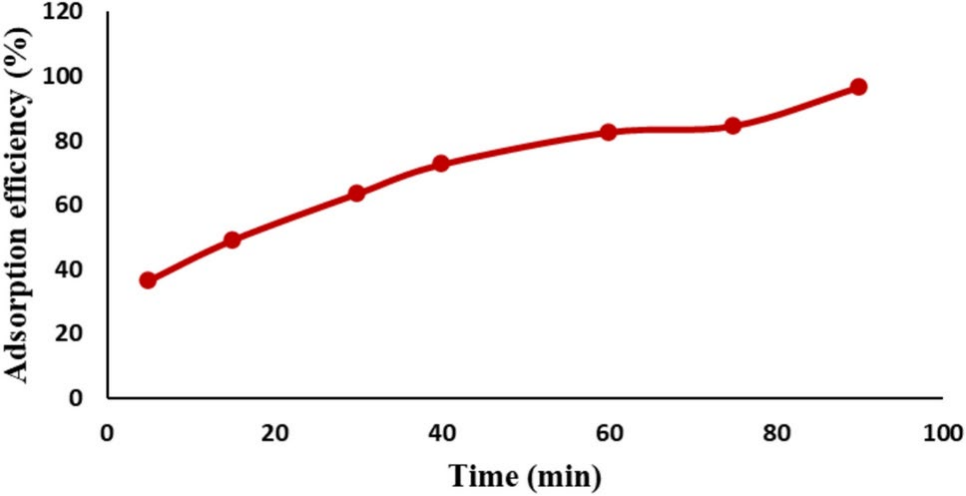
Effect of contact time on the adsorption efficiency of Remazol Red dye.

The adsorption of Remazol Red dye onto NiFe_2_O_4_ NPs surface was monitored to determine the time needed to reach equilibrium. Figure 11 illustrates how the dye removal efficiency changes over time. The adsorption rate increased steadily as the contact time progressed, eventually reaching equilibrium after 90 min. At equilibrium, the adsorption efficiency was recorded at 96.5 ± 0.4%, corresponding to an adsorption capacity of 19.3 ± 0.6 mg/g. This demonstrates that the nickel ferrite nanoparticles achieved rapid equilibrium during the removal of Remazol Red dye, highlighting their efficiency as an adsorbent.

#### Effect of dye concentration

**Fig. 12:**
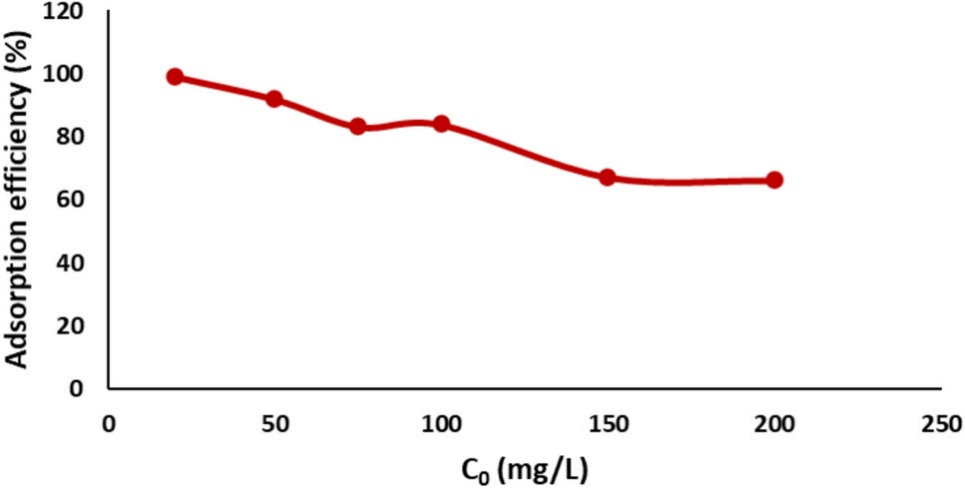
Dye concentration effect on the adsorption efficiency of Remazol Red dye.

Figure 12 displays the effect of varying the initial concentration of Remazol Red dye on the adsorption efficiency of NiFe_2_O_4_ nanoparticles. The experiments were conducted with initial dye concentrations ranging from 20 to 200 mg/L, a nickel ferrite dose of 1 g/L, pH 2, and a contact time of 90 min at 25 °C. The chosen concentration range was determined by its significance to standard dye concentrations observed in textile industry wastewater, as documented in previous research. These concentrations typically range from 10 to 250 mg/L, contingent upon the dyeing procedure and effluent dilution.^8,54^ This range enables the assessment of adsorption efficacy spanning low to moderately high dye concentrations, maintaining relevance to practical situations while permitting the calculation of the maximum adsorption capacity (q_max_) of the NiFe_2_O_4_ nanoparticles. The data reveal that there is a consistent decrease in dye removal percentage as the initial dye concentration increases. Specifically, at 20 mg/L, the removal efficiency was 99 ± 0.5%, decreasing to 91.8 ± 0.7% at 50 mg/L, 83.7 ± 0.5% at 100 mg/L, 67 ± 0.5% at 150 mg/L, and 66 ± 0.4% at 200 mg/L. At lower concentrations, the number of dye molecules is relatively small compared to the available active adsorption sites on the surface of the nanoparticles, leading to higher adsorption efficiency.^57^ The sufficient availability of active sites allows for compelling interactions and collisions between dye molecules and the adsorbent surface, resulting in near-complete removal of the dye. As the initial dye concentration increases, the number of dye molecules exceeds the available active sites on the surface of the nickel ferrite. This saturation of active sites reduces the probability of effective adsorption and decreases the removal efficiency. Furthermore, the competition among dye molecules for the limited active sites becomes more pronounced at higher concentrations, further lowering the dye removal percentage. This behavior highlights the importance of maintaining an appropriate adsorbent-to-dye ratio to achieve optimal removal efficiency.^58^

### Adsorption kinetics

#### Pseudo-first-order model

**Fig. 13:**
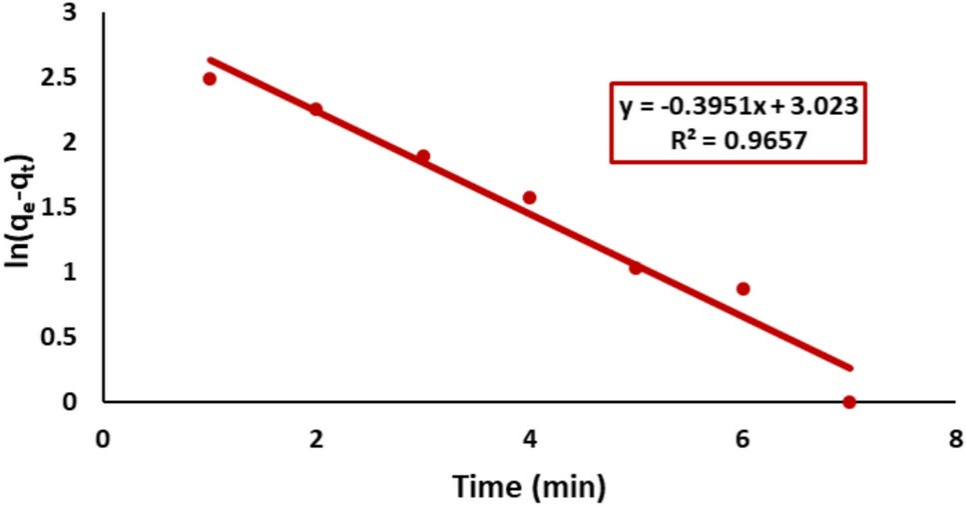
Pseudo first order plot for kinetic modeling of Remazol Red adsorbed onto nickel ferrite NPs.

The pseudo-first-order model was applied to analyze the adsorption kinetics of Remazol Red dye onto nickel ferrite. The rate constant K_1_ values for the adsorption of Remazol Red on nickel ferrite was determined from the plot of ln (q_e_ − q_t_) versus time yielding a high correlation (R^2^ = 0.96), suggesting a good fit to this model, as shown in Fig. 13.

#### Pseudo-second-order model

**Fig. 14:**
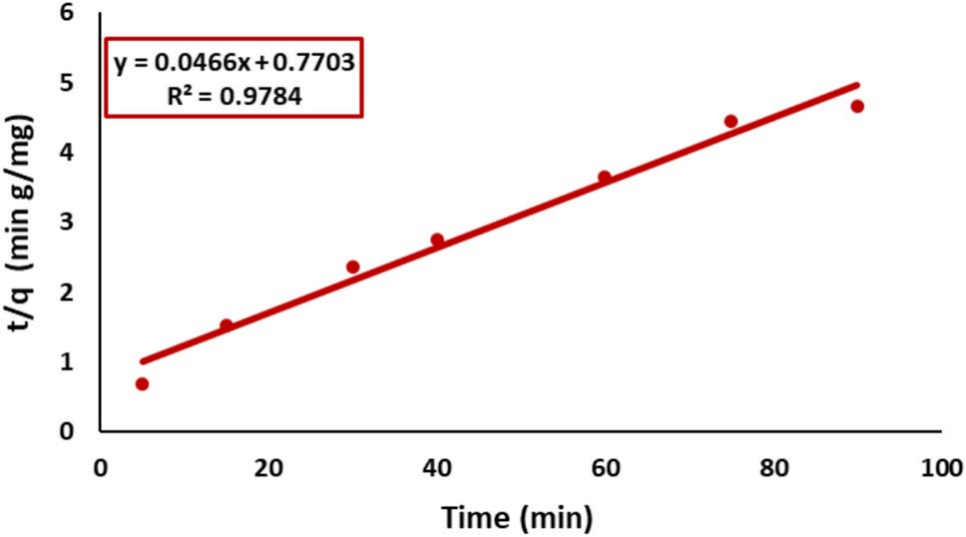
Pseudo second order plot for kinetic modeling of Remazol Red dye adsorption onto nickel ferrite NPs.

The pseudo-second-order model was also evaluated. Linear graphs of t/qt against time, Fig. 14 yielded the rate constant K_2_ and q_e_. The pseudo-second-order model, with a correlation coefficient (R^2^) of 0.978, demonstrates a superior fit relative to the pseudo-first-order model (R^2^ = 0.965). The principal factor influencing the model’s applicability is comparing the estimated and empirically obtained equilibrium adsorption capacity (qe) values.

The batch kinetic data were evaluated using pseudo-first-order and pseudo-second-order models, which accurately characterized the adsorption process with a 95% confidence level. The kinetic parameters, along with the calculated initial sorption rate values, are summarized in Table 1. The connection between estimated equilibrium adsorption capacities (q_e_) and experimental values indicates that the Pseudo-first-order model is a better fit than the Pseudo-second-order model.

**Table 1:** Kinetic parameters of Remazol Red dye adsorption over nickel ferrite.

*q*_*e(exp.)*_ (mg/g)	Pseudo-first-order			Pseudo-second-order			Weber–Morris		
	q_e_(cal.) (mg/g)	K_1_ (min^−1^)	R^2^	q_e_(cal.) (mg/g)	K_2_ (g/(mg min))	R^2^	K_d_	I	R^2^
19.3 ± 0.6	20.5 ± 0.8	0.39	0.96	21.4 ± 0.7	0.002	0.97	1.6	3.7	0.98

#### Weber–Morris kinetic model

**Fig. 15:**
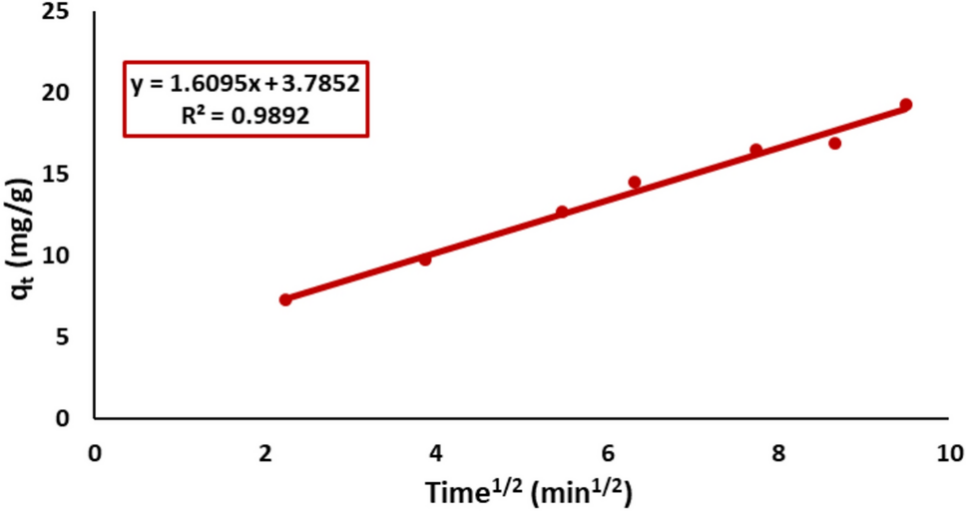
Weber–Morris for kinetic modeling of Remazol Red dye adsorption onto nickel ferrite NPs.

Regression analysis was performed on the graph illustrating the relationship between the quantity of Remazol Red dye adsorbed (q_t_) and the square root of time (t^1/2^) to assess the significance of diffusion in the sorption process. As illustrated in Fig. 15, the plot of qt versus t^1/2^ exhibits a linear relationship, indicating the involvement of intra-particle diffusion plays a role in the adsorption process of Remazol Red dye.

#### Boyd plot

**Fig. 16:**
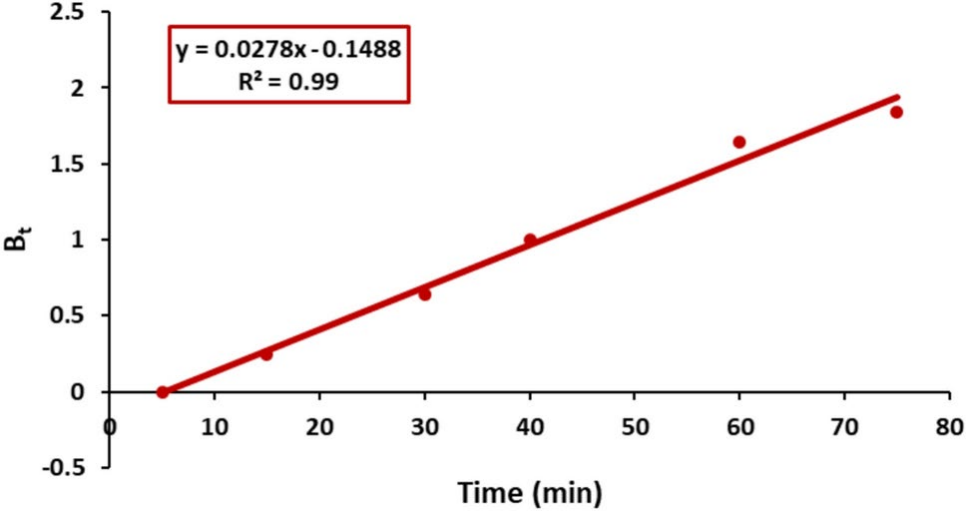
B_t_ versus time for Remazol Red dye adsorption onto nickel ferrite nanoparticles.

To confirm exactly the adsorption mechanism of Remazol Red dye on nickel ferrite the Boyd plot was investigated.^59^ Figure 16 illustrates the time profile of the calculated B_t_ values. The linearity of the plots provides valuable information for distinguishing between external mass transfer and intra-particle diffusion-controlled mechanisms of adsorption. It was observed that the plots did not pass through the origin, indicating the involvement of external mass transfer throughout the adsorption process. Thus, both intra-particle diffusion and external mass transfer contribute to the adsorption of Remazol Red onto nickel ferrite.

### Adsorption isotherms

#### Langmuir isotherm model

**Fig. 17:**
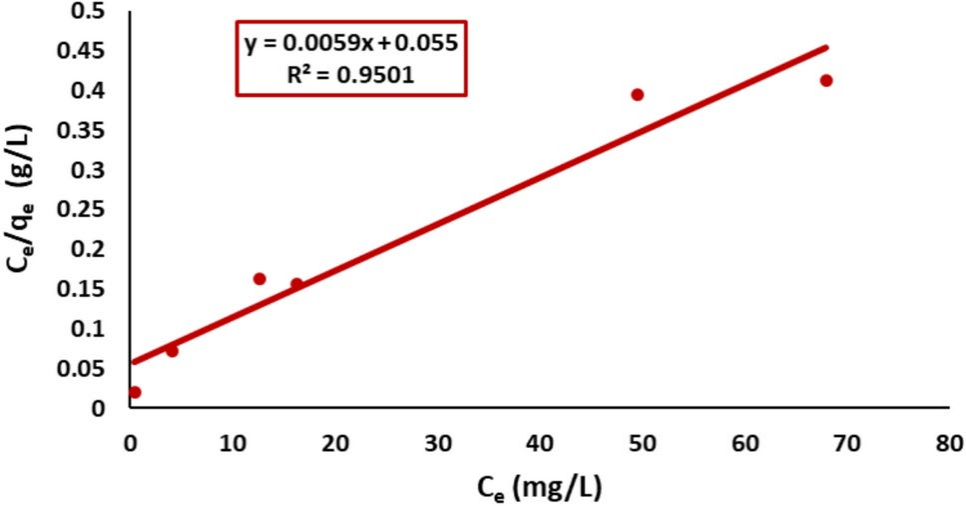
Langmuir plot of Remazol Red dye adsorption onto nickel ferrite nanoparticles.

The Langmuir isotherm constants were obtained from the plot of Ce/qe versus Ce. Table 3 summarizes the estimated isothermal constants and their accompanying correlation coefficients. The figure displays a linear relationship with R^2^ = 0.95. The maximum adsorption capacity (qmax) was determined to be 169.5 ± 0.8 mg/g, as shown in Fig. 17.

The isotherm’s form has been analyzed to evaluate the favorability of the adsorption process.^60^ The fundamental characteristic of Langmuir isotherms is represented by R_L_, a dimensionless constant known as the separation factor or equilibrium parameter.^61^ R_L_ is calculated using the following equation:

12 $${{\varvec{R}}}_{{\varvec{L}}}=\frac{1}{1+{{\varvec{K}}}_{{\varvec{L}}}{{\varvec{C}}}_{0}}$$

**Fig. 18:**
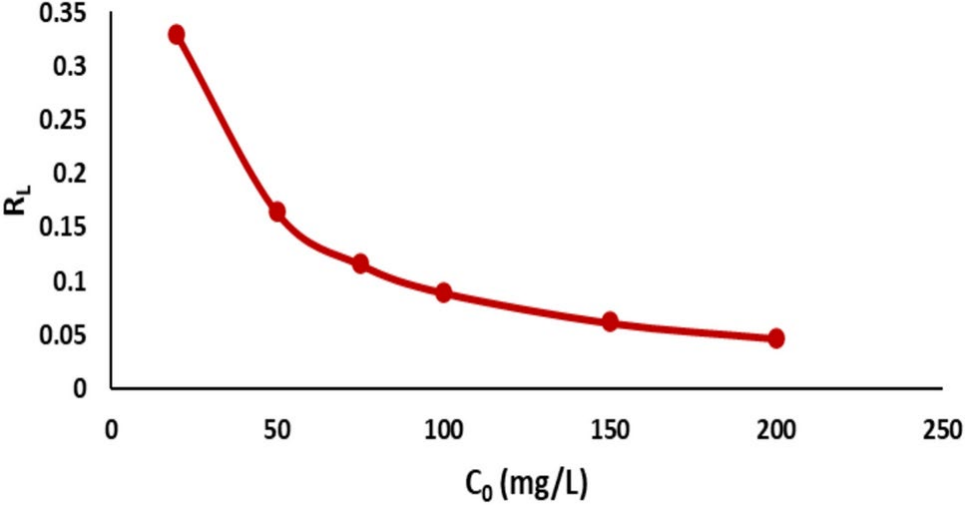
Variation of the separation factor (RL) with initial concentration for the adsorption of Remazol Red dye onto nickel ferrite nanoparticles.

The equilibrium parameter R_L_ in this study ranged from 0 to 1, indicating that the sorption process was favorable and the adsorbent showed significant potential for the sorption of Remazol Red ions, as shown in Fig. 18. A comparative analysis of the adsorption capacity of nickel ferrite with other materials for Remazol Red dye removal is presented in Table 2. The results indicate that nickel ferrite exhibits strong adsorption capabilities, second only to activated carbon.

**Table 2:** A comparison of the adsorption capacities of Remazol Red dye across various adsorbent materials.

Adsorbent	q_max_ (mg/g)	Refs.
Jute Stick Charcoal	93.12 mg/g	^62^
Treated Sawdust	50.0 mg/g	^63^
Activated Carbon from Walnut Shell Biomass	54.38 mg/g	^64^
Poly (acrylamide-co-acrylic acid) Hydrogel	44.19 mg/g	^65^
Activated carbon (600–700 μm)	213 mg/g	^66^
Bagasse fly ash	18.2 mg/g	^67^
Eggshell biocomposite	46.9 mg/g	^54^
Nickel ferrite NPs	169.5 ± 0.8 mg/g	Current work

#### Freundlich isotherm model

**Fig. 19:**
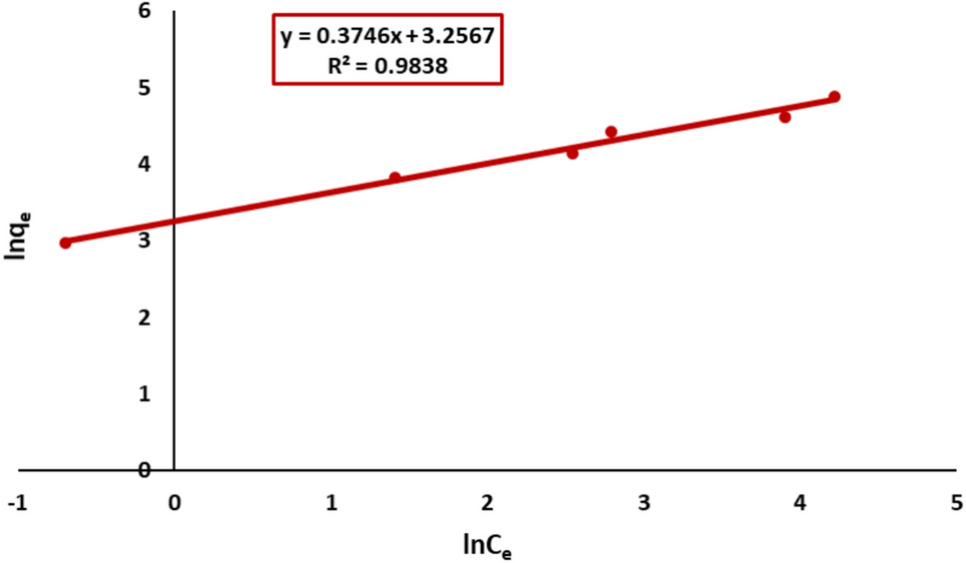
Freundlich plot of Remazol Red dye adsorption onto nickel ferrite nanoparticles.

The Freundlich model showed a stronger fit (R^2^ = 0.98) from the plot of ln qe versus ln Ce suggesting multilayer adsorption on a heterogeneous surface as shown in Fig. 19. The estimated isotherm parameters and their correlation coefficients are presented in Table 3. Both the Langmuir and Freundlich isotherm models demonstrated statistical significance at a 95% confidence level. The adsorption of Remazol Red dye onto nickel ferrite exhibited a strong correlation with the Freundlich equation (R^2^ > 0.98), surpassing the correlation with the Langmuir equation (R^2^ > 0.95) within the examined concentration range. Consequently, the Freundlich isotherm demonstrates superior fit relative to the Langmuir isotherm across all situations, as seen by the correlation coefficients R^2^.

**Table 3:** Adsorption isotherms constants and correlation coefficients.

Model	Parameter	Value
Langmuir	q_max_ mg/g	169.5
	K_L_ L/mg	0.107
	R^2^	0.95
Freundlich	K_f_ mg/g	25.9
	1/n	0.37
	R^2^	0.98
Temkin	K_T_ L/g	3.13
	B_T_ J/mol	21.12
	R^2^	0.9
Dubinin–Radushkevich	β mol^2^/J^2^	3 × 10^–9^
	q_DR_ mg/g	496
	E KJ/mol	12.9
	R^2^	0.98

The adsorption equilibrium data were more fitted by the Freundlich model compared to the Langmuir model, indicating that multilayer adsorption took place on a heterogeneous surface. This discovery suggests that the adsorption sites on NiFe_2_O_4_ nanoparticles were heterogeneous, resulting in discrepancies in adsorption energy.

#### Temkin isotherm model

**Fig. 20:**
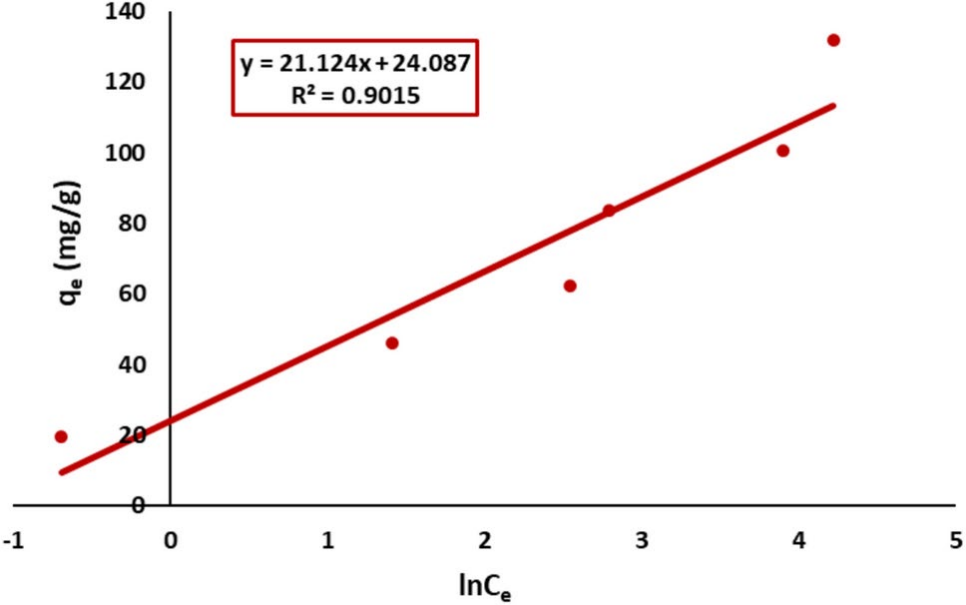
Temkin plot of Remazol Red dye adsorption onto nickel ferrite nanoparticles.

The Temkin model was evaluated via the plot of qe versus lnCe reflecting a linear decrease in sorption heat with coverage that fits pretty well (R^2^ = 0.90), as shown in Fig. 20.

#### Dubinin–Radushkevich isotherm model

**Fig. 21:**
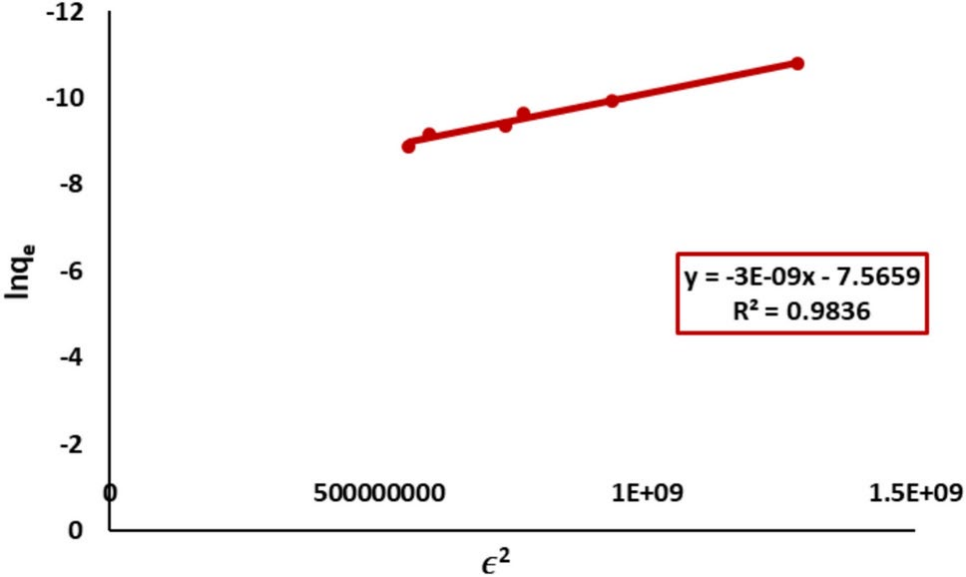
D-R plot of Remazol Red dye adsorption onto nickel ferrite nanoparticles.

The Dubinin-Radushkevich (D-R) model was analyzed using the plot of ln qe versus ε^2^, Fig. 21. The adsorption energy (E = 12.9 kJ/mol) suggested a mix of physisorption and weak chemisorption. The D-R isotherm parameters are displayed in Table 2.

## Recyclability of magnetic nickel ferrite for Remazol Red dye removal

**Fig. 22:**
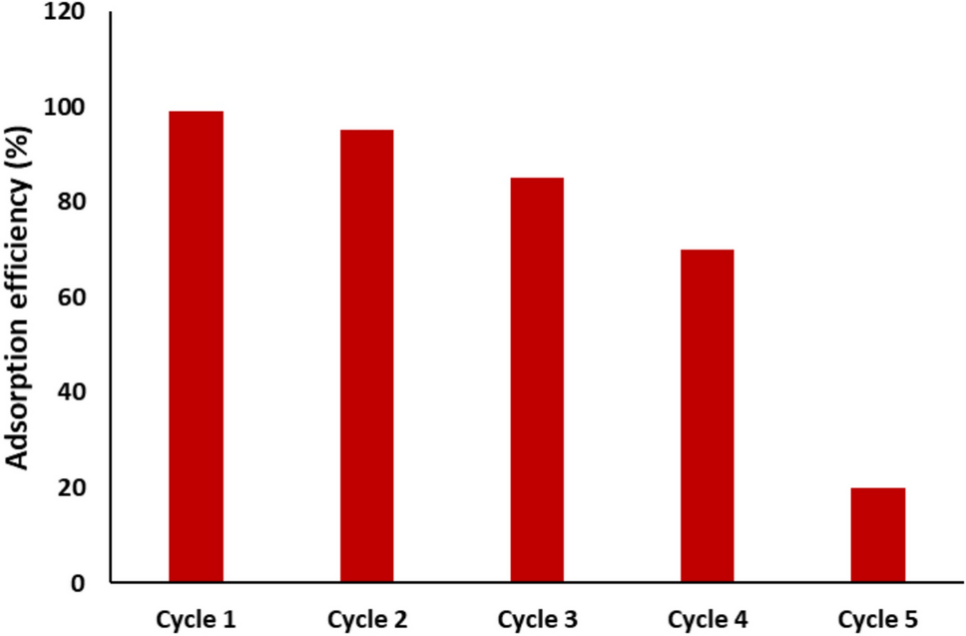
Recyclability of magnetic Nickel Ferrite NPs for Remazol Red dye removal.

The recyclability of the synthesized magnetic nickel ferrite nanoparticles was evaluated by conducting five consecutive adsorption–desorption cycles under consistent optimal conditions, including a pH of 2, a contact time of 90 min, and an initial Remazol Red dye concentration of 20 mg/L. After each cycle, the used nanoparticles were recovered, thoroughly washed with distilled water and ethanol to remove residual dye, and then dried before being reused in the next cycle. As depicted in Fig. 22, the adsorption efficiency of the nanoparticles showed a progressive decline over the five cycles. Initially, the adsorption efficiency was recorded at an impressive 99 ± 1.1% during the first cycle, demonstrating the material’s strong initial dye-removal capability. However, the efficiency decreased significantly with each successive cycle, dropping to 20 ± 0.7% by the fifth cycle. This reduction in efficiency could be attributed to the gradual loss of active adsorption sites on the surface of the nanoparticles due to fouling or incomplete desorption of the dye molecules.^16^ Additionally, structural or morphological changes in the nanoparticles during repeated use, such as aggregation or partial degradation, might have contributed to this decline in performance.

## Conclusion

Magnetic nickel ferrite (NiFe_2_O_4_) nanoparticles, synthesized via a facile, low-temperature aqueous reflux method without calcination, effectively removed 99 ± 1.1% of Remazol Red dye from wastewater after 90 min under optimal conditions (pH 2, 1 g/L dose, 20 mg/L dye concentration). Adsorption efficiency increased with lower pH and higher dosage, driven by the nanoparticles’ properties. Characterization confirmed a crystalline cubic spinel structure, 23 nm average particle size, and superparamagnetic behavior (56.96 ± 0.9 emu/g), enhancing surface area and separability. Kinetic studies aligned with pseudo-first-order, Weber–Morris, and Boyd models (R^2^ = 0.96), indicating surface interactions and mixed external mass transfer/intra-particle diffusion mechanisms. The Freundlich isotherm (R^2^ = 0.98) and a maximum adsorption capacity of 169.5 ± 0.8 mg/g suggest multilayer physisorption on a heterogeneous surface, with the PZC (6.7) explaining higher efficiency at pH < 6.7 due to electrostatic attraction between the positively charged surface and anionic dye, and repulsion above PZC. The Dubinin–Radushkevich model (E = 12.9 kJ/mol) hints at minor chemisorption, though physisorption dominates. Reusability tests showed a decline from 99 ± 01.1% to 20 ± 0.7% over five cycles, highlighting stability challenges. This study demonstrates NiFe_2_O_4_ as a cost-effective, sustainable adsorbent, with a mechanism blending physisorption (via van der Waals and electrostatic forces) and weak chemisorption. Future work should focus on stabilizing recyclability through surface modifications and testing multi-pollutant systems to mimic real wastewater, alongside exploring greener synthesis routes.

## Data Availability

The datasets generated and/or analysed during the current study are not yet publicly available as they are being prepared for deposition but are available from the corresponding author (Yosri.fahim@gu.edu.eg) on reasonable request.

## References

[1] Rahimi, S. M. et al. Application of CuFe_2_O_4_/CuS as a new green magnetic nanocomposite in adsorption of tetracycline from aqueous solutions: Mathematical models of thermodynamics, isotherms, and kinetics. *Appl. Water Sci.* **15**(1), 1–17 (2025).

[2] Hossein Panahi, A. et al. Photocatalytic degradation of humic acid using bentonite@ Fe_3_O_4_@ZnO magnetic nanocomposite: An investigation of the characterization of the photocatalyst, degradation pathway, and modeling by solver plugin. *Water* **15**(16), 2931 (2023).

[3] Fahim, Y.A. et al., *Occupational Exposure to Heavy Metal Dust and Its Hazardous Effects on Non-ferrous Foundry Workers’ Health* (2024).

[4] Mittal, R. et al. Surface modified novel synthesis of Spirulina assisted mesoporous TiO_2_@ CTAB nanocomposite employed for efficient removal of chromium (VI) in wastewater. *Appl. Surf. Sci.* **679**, 161309 (2025).

[5] Patar, S. et al. Algae derived N-doped mesoporous carbon nanoflakes fabricated with nickel ferrite for photocatalytic removal of Congo Red and Rhodamine B dyes. *Surfaces Interfaces* **51**, 104710 (2024).

[6] Nanjani, S., & Keharia, H. K. Alterations in microbial community structure and function in response to Azo Dyes. In *Microbiome-Host Interactions*.CRC Press 367–395 (2021).

[7] Hashemi, S.H., & Kaykhaii, M. Azo dyes: Sources, occurrence, toxicity, sampling, analysis, and their removal methods. In *Emerging Freshwater Pollutants*. 267–287. Elsevier (2022).

[8] Al-Tohamy, R. et al. A critical review on the treatment of dye-containing wastewater: Ecotoxicological and health concerns of textile dyes and possible remediation approaches for environmental safety. *Ecotoxicol. Environ. Saf.* **231**, 113160 (2022). 35026583 10.1016/j.ecoenv.2021.113160

[9] Zhao, C. et al. Application of coagulation/flocculation in oily wastewater treatment: A review. *Sci. Total Environ.* **765**, 142795 (2021). 33572034 10.1016/j.scitotenv.2020.142795

[10] Lu, X., & Liu, R. Treatment of azo dye-containing wastewater using integrated processes. *Biodegradation of azo dyes*, 133–155 (2010).

[11] Teh, C. Y. et al. Recent advancement of coagulation–flocculation and its application in wastewater treatment. *Ind. Eng. Chem. Res.* **55**(16), 4363–4389 (2016).

[12] De Gisi, S. et al. Characteristics and adsorption capacities of low-cost sorbents for wastewater treatment: A review. *Sustain. Mater. Technol.* **9**, 10–40 (2016).

[13] Al-Musawi, T. J. et al. Hexadecyltrimethylammonium-activated and zinc oxide-coated nano-bentonite: A promising photocatalyst for tetracycline degradation. *Sustain. Energy Technol. Assess.* **53**, 102451 (2022).

[14] Nasseh, N. et al. Photo-catalytic degradation of tamoxifen by using a novel synthesized magnetic nanocomposite of FeCl_2_@ ac@ ZnO: A study on the pathway, modeling, and sensitivity analysis using artificial neural network (AAN). *J. Environ. Chem. Eng.* **10**(3), 107450 (2022).

[15] Fahim, Y. A. et al. A review on Lipases: Sources, assays, immobilization techniques on nanomaterials and applications. *BioNanoScience* **14**(2), 1780–1797 (2024).

[16] El-Khawaga, A. M. et al. Promising photocatalytic and antimicrobial activity of novel capsaicin coated cobalt ferrite nanocatalyst. *Sci. Rep.* **13**(1), 5353 (2023). 37005443 10.1038/s41598-023-32323-y PMC10067836

[17] Azqandi, M. et al. Green synthesis of manganese ferrite magnetic nanoparticle and its modification with metallic-organic frameworks for the tetracycline adsorption from aqueous solutions: A mathematical study of kinetics, isotherms, and thermodynamics. *Environ. Res.* **256**, 118957 (2024). 38636645 10.1016/j.envres.2024.118957

[18] Hassan, A. A., Fahim, Y. A. & Ali, M. E. M. Efficient removal of Cr (VI) and As (V) from aqueous solution using magnetically separable nickel ferrite nanoparticles. *J. Cluster Sci.* **36**(1), 1–18 (2025). 10.1038/s41598-025-98478-y PMC12092790 40394334

[19] Granados-Miralles, C. & Jenuš, P. On the potential of hard ferrite ceramics for permanent magnet technology—A review on sintering strategies. *J. Phys. D Appl. Phys.* **54**(30), 303001 (2021).

[20] Starko, I., Tatarchuk, T. & Naushad, M. The potential of Gd doping as a promising approach for enhancing the adsorption properties of nickel–cobalt ferrites. *Environ. Sci. Pollut. Res.* **31**(43), 55462–55474 (2024). 10.1007/s11356-024-34809-2 39230814

[21] Fathy, M. A., Kamel, A. H. & Hassan, S. S. M. Novel magnetic nickel ferrite nanoparticles modified with poly (aniline-*co*-o-toluidine) for the removal of hazardous 2,4-dichlorophenol pollutant from aqueous solutions. *RSC Adv.* **12**(12), 7433–7445 (2022). 35424706 10.1039/d2ra00034b PMC8982154

[22] Taj, M. B. et al. Bioconjugate synthesis, phytochemical analysis, and optical activity of NiFe_2_O_4_ nanoparticles for the removal of ciprofloxacin and Congo red from water. *Sci. Rep.* **11**(1), 5439 (2021). 33686116 10.1038/s41598-021-84983-3 PMC7970886

[23] Patar, S. et al. Photocatalytic degradation of antibiotics by N-doped carbon nanoflakes-nickel ferrite composite derived from algal biomass. *Chemosphere* **363**, 142908 (2024). 39033863 10.1016/j.chemosphere.2024.142908

[24] Sivakumar, P. et al. Preparation and properties of nickel ferrite (NiFe_2_O_4_) nanoparticles via sol–gel auto-combustion method. *Mater. Res. Bull.* **46**(12), 2204–2207 (2011).

[25] Patra, J. K. et al. Nano based drug delivery systems: Recent developments and future prospects. *J. Nanobiotechnol.* **16**(1), 1–33 (2018). 10.1186/s12951-018-0392-8 PMC6145203 30231877

[26] Aditha, S. K. et al. Aqueous based reflux method for green synthesis of nanostructures: Application in CZTS synthesis. *MethodsX* **3**, 35–42 (2016). 27408826 10.1016/j.mex.2015.12.003 PMC4929250

[27] Nazibudin, N. A. et al. The application of endoscope and image processing for determining Remazol Red dye concentration in water samples. *Jurnal Teknologi* **84**(6), 115–123 (2022).

[28] Rahimi, S. M. et al. Magnetically recoverable nickel ferrite coated with CuS nanocomposite for degradation of metronidazole in photocatalytic and photo Fenton like processes. *Int. J. Environ. Anal. Chem.* **102**(18), 6699–6719 (2022).

[29] Mazari Moghaddam, N. S. et al. Effective removal of Sirius yellow K-CF dye by adsorption process onto chitosan-polyacrylamide composite loaded with ZnO nanoparticles. *Int. J. Environ. Anal. Chem.* **103**(20), 8782–8798 (2023).

[30] Chern, J.-M. & Wu, C.-Y. Desorption of dye from activated carbon beds: Effects of temperature, pH, and alcohol. *Water Res.* **35**(17), 4159–4165 (2001). 11791845 10.1016/s0043-1354(01)00127-0

[31] Mittal, A. et al. Studies on the adsorption kinetics and isotherms for the removal and recovery of Methyl Orange from wastewaters using waste materials. *J. Hazard. Mater.* **148**(1–2), 229–240 (2007). 17379402 10.1016/j.jhazmat.2007.02.028

[32] Viegas, R. M. C. et al. How do the HSDM and Boyd’s model compare for estimating intraparticle diffusion coefficients in adsorption processes. *Adsorption* **20**, 737–746 (2014).

[33] Ghosal, P. S. & Gupta, A. K. Development of a generalized adsorption isotherm model at solid–liquid interface: A novel approach. *J. Mol. Liq.* **240**, 21–24 (2017).

[34] Barkhor, H. et al., Investigating mathematical models for the surface adsorption process of tetracycline antibiotic using a CuFe_12_O_19_/CuS green magnetic nanocomposite*.* In *Biomass Conversion and Biorefinery* (2024).

[35] Desta, M. B. Batch sorption experiments: Langmuir and Freundlich isotherm studies for the adsorption of textile metal ions onto teff straw (*Eragrostis tef*) agricultural waste. *J. Thermodyn.* **2013**(1), 375830 (2013).

[36] Obaid, S.A. *Langmuir, Freundlich and Tamkin adsorption isotherms and kinetics for the removal aartichoke tournefortii straw from agricultural waste*. IOP Publishing.

[37] Sampranpiboon, P., Charnkeitkong, P. & Feng, X. Equilibrium isotherm models for adsorption of zinc (II) ion from aqueous solution on pulp waste. *WSEAS Trans. Environ. Dev.* **10**, 35–47 (2014).

[38] Puccia, V. & Avena, M. J. On the use of the Dubinin-Radushkevich equation to distinguish between physical and chemical adsorption at the solid-water interface. *Colloid Interface Sci. Commun.* **41**, 100376 (2021).

[39] Akyol, A. & Bayramoglu, M. The degradation of an azo dye in a batch slurry photocatalytic reactor. *Chem. Eng. Process.* **47**(12), 2150–2156 (2008).

[40] Khairy, M. et al. Kinetics and isotherm studies of Remazol Red adsorption onto polyaniline/cerium oxide nanocomposites. *J. Basic Environ. Sci.* **3**(4), 123–132 (2016).

[41] Hosni, N. et al. Synthesis of (2D) MNPs nanosheets of nickel ferrite using a low-cost co-precipitation process. *Mater. Sci. Eng. B* **232**, 48–54 (2018).

[42] Huo, J. & Wei, M. Characterization and magnetic properties of nanocrystalline nickel ferrite synthesized by hydrothermal method. *Mater. Lett.* **63**(13–14), 1183–1184 (2009).

[43] Starko, I., Tatarchuk, T., & Bououdina, M. La-doped Ni_0.5_Co_0.5_Fe_2_O_4_ nanoparticles: Effect of cobalt precursors on structure and morphology. In *Molecular Crystals and Liquid Crystals* (2018).

[44] Fahim, Y. A. et al. Immobilized lipase enzyme on green synthesized magnetic nanoparticles using *Psidium guava* leaves for dye degradation and antimicrobial activities. *Sci. Rep.* **14**(1), 8820 (2024). 38627424 10.1038/s41598-024-58840-y PMC11021406

[45] Branca, C. et al. Role of the OH and NH vibrational groups in polysaccharide-nanocomposite interactions: A FTIR-ATR study on chitosan and chitosan/clay films. *Polymer* **99**, 614–622 (2016).

[46] Goldman, A., *Ferrites for magnetic Recording.* In *Modern ferrite technology*, 353–373 (2006).

[47] Sezer, N. et al. Superparamagnetic nanoarchitectures: Multimodal functionalities and applications. *J. Magn. Magn. Mater.* **538**, 168300 (2021).

[48] Naseri, M. G. et al. Simple preparation and characterization of nickel ferrite nanocrystals by a thermal treatment method. *Powder Technol.* **212**(1), 80–88 (2011).

[49] Nandapure, A. I. et al. Effect of zinc substitution on magnetic and electrical properties of nanocrystalline nickel ferrite synthesized by refluxing method. *Physica B* **407**(7), 1104–1107 (2012).

[50] Terzyk, A. P. et al. New correlations between the composition of the surface layer of carbon and its physicochemical properties exposed while paracetamol is adsorbed at different temperatures and pH. *J. Colloid Interface Sci.* **257**(1), 13–30 (2003).

[51] Nasiruddin Khan, M. & Sarwar, A. Determination of points of zero charge of natural and treated adsorbents. *Surface Rev. Lett.* **14**(03), 461–469 (2007).

[52] Amulya, M. A. S. et al. Sonochemical synthesis of NiFe2O4 nanoparticles: Characterization and their photocatalytic and electrochemical applications. *Appl. Surface Sci. Adv.* **1**, 100023 (2020).

[53] El Qada, E. N., Allen, S. J. & Walker, G. M. Adsorption of methylene blue onto activated carbon produced from steam activated bituminous coal: A study of equilibrium adsorption isotherm. *Chem. Eng. J.* **124**(1–3), 103–110 (2006).

[54] Elkady, M. F., Ibrahim, A. M. & Abd El-Latif, M. M. Assessment of the adsorption kinetics, equilibrium and thermodynamic for the potential removal of reactive red dye using eggshell biocomposite beads. *Desalination* **278**(1–3), 412–423 (2011).

[55] Rápó, E. et al. Adsorption of remazol brilliant violet-5R textile dye from aqueous solutions by using eggshell waste biosorbent. *Sci. Rep.* **10**(1), 8385 (2020). 32433528 10.1038/s41598-020-65334-0 PMC7239865

[56] Badawi, A. K., Abd Elkodous, M. & Ali, G. A. M. Recent advances in dye and metal ion removal using efficient adsorbents and novel nano-based materials: An overview. *RSC Adv.* **11**(58), 36528–36553 (2021). 35494372 10.1039/d1ra06892j PMC9043615

[57] Tan, K. B. et al. Adsorption of dyes by nanomaterials: Recent developments and adsorption mechanisms. *Sep. Purif. Technol.* **150**, 229–242 (2015).

[58] Etim, U. J., Umoren, S. A. & Eduok, U. M. Coconut coir dust as a low cost adsorbent for the removal of cationic dye from aqueous solution. *J. Saudi Chem. Soc.* **20**, S67–S76 (2016).

[59] Komal, et al., Spinel nanoferrites: A versatile platform for environmental remediation. In *Spinel Nanoferrites: Synthesis, Properties and Applications*, 315–347 (2021).

[60] Wang, J. & Guo, X. Adsorption isotherm models: Classification, physical meaning, application and solving method. *Chemosphere* **258**, 127279 (2020). 32947678 10.1016/j.chemosphere.2020.127279

[61] Saleh, T.A. Isotherm models of adsorption processes on adsorbents and nanoadsorbents. In *Interface Science and Technology* 99–126. Elsevier (2022).

[62] Uddin, A. N. et al. Adsorptive removal of Remazol Red (RR) from textile effluents using jute stick charcoal (JSC). *H2Open J.* **7**(1), 78–92 (2024).

[63] Ara, N. J. et al. Removal of remazol red from textile waste water using treated sawdust—An effective way of effluent treatment. *Bangladesh Pharm. J.* **16**(1), 93–98 (2013).

[64] Parimelazhagan, V. et al. Rapid removal of toxic Remazol brilliant blue-R dye from aqueous solutions using *Juglans nigra* shell biomass activated carbon as potential adsorbent: Optimization, isotherm, kinetic, and thermodynamic investigation. *Int. J. Mol. Sci.* **23**(20), 12484 (2022). 36293336 10.3390/ijms232012484 PMC9604326

[65] Corona-Rivera, M. A. et al. Remazol red dye removal using poly (acrylamide-*co*-acrylic acid) hydrogels and water absorbency studies. *Colloid Polym. Sci.* **295**, 227–236 (2017).

[66] Al-Degs, Y. et al. Effect of carbon surface chemistry on the removal of reactive dyes from textile effluent. *Water Res.* **34**(3), 927–935 (2000).

[67] Rachakornkij, M., Ruangchuay, S. & Teachakulwiroj, S. Removal of reactive dyes from aqueous solution using bagasse fly ash. *Songklanakarin J. Sci. Technol.* **26**(1), 13–24 (2004).

